# Supranutritional Selenomethionine but not Selenite Reduces Malignant Cell Transformation

**DOI:** 10.1007/s12011-025-04719-6

**Published:** 2025-06-25

**Authors:** Caroline E. Meyer, Maria Schwarz, Felix B. Meyer, René Thierbach, Anna P. Kipp

**Affiliations:** 1 Department of Nutritional Physiology, Institute of Nutritional Sciences, Friedrich Schiller University Jena, Dornburger Str. 24, 07743 Jena, Germany; 2 Human Nutrition, Institute of Nutritional Sciences, Friedrich Schiller University Jena, Jena, Germany

**Keywords:** Malignant cell transformation, Selenium, Selenomethionine, Selenite

## Abstract

The trace element selenium is essential to human health and has long been discussed to modulate the carcinogenic process. So far, the functions of selenium and selenoproteins have been mainly studied in already transformed cancer cells. The aim of this study was, therefore, to characterize the role of selenium during the neoplastic transformation process in a concentration- and species-specific manner using the BALB/c cell transformation assay. This assay mimics the initiation and promotion phase of BALB/c cells (murine fibroblasts) treated with a tumor initiator (MCA) and promoter (TPA) leading to malignant cell foci development. In parallel, cells were supplied with selenite or selenomethionine using suboptimal, adequate or supranutritional concentrations (0.01—1 μM). The supranutritional selenomethionine concentration reduced malignantly transformed foci, accompanied by higher NQO1 levels suggesting an upregulation of antioxidant NRF2 target genes. In contrast, treatment with the same concentration of selenite appeared to increase malignant transformation which was associated with higher intracellular selenium concentrations. Neither suboptimal nor adequate concentrations of either selenium species affected malignant cell transformation even though selenoprotein expression was modulated. Overall, the current data suggest that high concentrations of selenium have different effects on malignant cell transformation depending on the distinct selenium species applied.

**Supplementary Information:**

The online version contains supplementary material available at 10.1007/s12011-025-04719-6.

## Introduction

Cancer is a multifactorial disease and tumorigenesis a multistep process that is driven by genomic instability, enabling the progressive transformation of a normal cell into a malignant derivative [1]. Currently, one in five men or women develop cancer within their lifetime [2], emphasizing the need for successful prevention. Already in the 1970s, epidemiological studies showed that there is an inverse relationship between selenium intake and cancer mortality [3]. Also, results from the European Prospective Investigation into Cancer and Nutrition (EPIC) study revealed that a low selenium status is associated with an increased risk to develop colorectal [4] and hepatobiliary cancer [5].

Selenium is an essential trace element with diverse functions in human health [6]. Its biological effects are largely mediated by selenoproteins which contain the amino acid selenocysteine within their active center [6]. Most of the selenoproteins are oxidoreductases, catalyzing oxidation–reduction reactions, and thus are essentially involved in regulating the cellular redox status, affecting processes such as oxidative DNA damage but also the activity of redox-sensitive signaling pathways [6]. Accordingly, they have on the one hand the potential to protect a healthy cell from oxidative DNA damage, which would lower the amount of initiated tumor cells and result in tumor prevention. On the other hand, already established tumor cells need to combat higher basal levels of oxidative stress due to their higher metabolic rate [7]. Hence, those tumor cells are supposed to profit from an improved selenium supply and high expression levels of antioxidant selenoproteins. Accordingly, glutathione peroxidases (GPX) and thioredoxin reductase 1 (TXNRD1) are mostly upregulated in transformed cells [8, 9]. Consistent with this, the selenoproteins GPX2 and TXNRD1 have been shown to support tumor growth, but others, such as selenoprotein H (SELENOH), can suppress tumor cell proliferation [10]. Thus, selenoproteins modulate both cancer prevention and the promotion of established cancer cells [10].

However, there are also completely different approaches applying very high, supranutritional doses of selenium as antineoplastic or adjuvant agents as part of cancer therapy [11], potentially provoking direct effects of selenium or specific selenium species. High doses of selenium itself or in combination with chemotherapy and radiation have been described to be selectively cytotoxic to tumor cells and to inhibit tumor growth [11]. The specificity for tumor cells is achieved by their ability to accumulate selenium. Dietary selenium comprises different chemical forms including organic (*e.g.* selenomethionine) and inorganic species (*e.g.* selenite) [12]. While selenite is the most common species used for selenium supplements, selenomethionine is the main form taken up by food [12]. As potential therapeutic effects of selenium are not primarily mediated by selenoproteins but by selenocompounds, the chemical form of the selenium used is important in this context.

To date, cancer cell lines or tumor xenograft models have been mainly used to investigate the effects of selenium on tumor development, in which, however, malignant cell transformation has already occurred. To study the role of selenium during the whole neoplastic transformation process, we used the BALB/c cell transformation assay (BALB-CTA) as an *in vitro* tumorigenesis model. This assay has been successfully applied to understand molecular pathways involved in different stages of the transformation process. Analogous to *in vivo* multistage carcinogenesis, *in vitro* malignant cell transformation is described as a progressive process through qualitatively different stages involving similar cellular and molecular events [13, 14]. The phenomenon of cell transformation comprises phenotypic alterations (*e.g*., spindle-shaped morphology; basophilic staining), changes in growth behavior and control (*e.g*., immortality, multi-layered and anchorage independent growth) as well as tumorigenicity when transplanted into susceptible immune-compromised animals [15–17]. The BALB-CTA mimics the initiation and promotion phase of transformation [18]. The chemical induction of cellular transformation by a known tumor initiator (3-Methylcholanthrene, MCA) and promoter (12 O-Tetradecanoyl-phorbol-13-acetate, TPA) leads to the formation of morphological aberrant foci. A great benefit of this method is that both untransformed and transformed cells can be investigated in parallel.

To the best of our knowledge, selenium has not been studied in the BALB-CTA so far. Thus, the study aimed to characterize the role of selenium during the neoplastic transformation process in a concentration- and selenium species-specific manner. Using the BALB-CTA we were able to mimic the whole process of malignant cell transformation including the initiation phase by starting with healthy, untransformed cells. We added a pre-incubation phase, in which untransformed cells were supplemented with suboptimal (0.01 µM), adequate (0.1 µM), or supranutritional (1 µM) selenium concentrations to modulate the selenoprotein expression prior to tumor initiation. This allowed us to examine a preventive rather than a therapeutic effect of selenium on malignant cell transformation.

## Materials and Methods

### Cell Culture

**Fig. 1:**
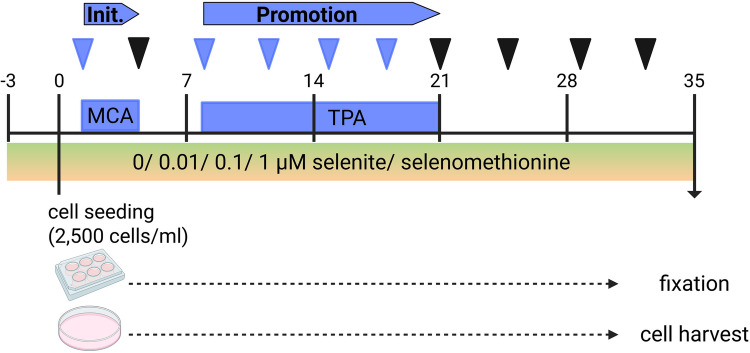
Scheme of the treatment conditions within the BALB-CTA. Untransformed BALB/c cells were treated without or with suboptimal, adequate, and supranutritional concentrations (0.01, 0.1, 1 µM) of selenite or selenomethionine for 72 h (pre-treatment, days −3 to 0). 2,500 cells/ml of pre-treated cells were seeded for the BALB-CTA. Cells were treated with a tumor initiator (0.5 µg/ml MCA; day 1 to 4) and afterwards with a tumor promoter (0.3 µg/ml TPA, days 8 to 21) or with DMSO as control. Additionally, cells were treated chronically with selenite or selenomethionine for the whole assay duration of 35 days. Cell culture media were replaced by fresh media every 3 to 4 days (symbolized by triangles). After 35 days, cells were fixed with methanol and stained with basophilic giemsa solution (6-well plates) or harvested (10-cm dishes) for further analyses

The murine embryonic fibroblast cell line BALB/c-3T3-A31-1–1 (BALB/c cells, Hatano Research Institute, Japan) was used for all experiments. Cells were cultured in DMEM/F12 medium (Pan Biotech, Aidenbach, Germany) supplemented with 5% fetal calf serum (FCS; Merck, Darmstadt, Germany) and 1% penicillin/streptomycin (P/S; Thermo Fisher Scientific, Waltham, Massachusetts, USA) under standard conditions (37 °C, 95% humidity, 5% CO_2_). The selenium content of the FCS was below the detection limit (< 2.6 µg/L), resulting in a selenium concentration of < 1.7 nM in the basal cell culture medium. Passaging of cells occurred twice a week at a confluency of 70–80% using trypsin/EDTA (Thermo Fisher Scientific). For experiments, cells were treated with sodium selenite (Thermo Fisher Scientific) or selenomethionine (Thermo Fisher Scientific) in the respective concentrations. For short-term experiments with a duration of 72 h, the cells were only treated once at the time point of cell seeding. For the BALB/c cell transformation assay (Fig. 1), cells were pre-treated with or without selenium for 72 h before they were seeded. During the 35 days of the experiment, medium with or without selenium was refreshed every 3–4 days.

Cells were harvested using trypsin/EDTA. Briefly, cell culture media were discarded, cells were washed with phosphate-buffered saline (PBS; 140 mM NaCl (Carl Roth), 10 mM Na_2_HPO_4_ (Carl Roth), 2.99 mM KH_2_PO_4_ (Carl Roth); pH 7.4) and incubated with trypsin/EDTA (Thermo Fisher Scientific) for 3–5 min at 37 °C. Fresh cell culture medium was added and cell suspensions were centrifuged for 3 min at room temperature (RT) (250 × *g*). Next, supernatants were discarded, and cells were washed with PBS and centrifuged for 3 min at RT (250 × *g*). Cell pellets were frozen in liquid nitrogen and stored at −20 °C until further preparation. In case of cells harvested from the BALB-CTA, cell suspensions were divided into two for subsequent preparations. Created in BioRender. Lossow, K. (2025) https://BioRender.com/9tu7eu3. 

### BALB/c Cell Transformation Assay

In the classical *in vitro* assay BALB-CTA, BALB/c cells are treated with the tumor initiator MCA and tumor promoter TPA leading to chemical transformation of cells during a period of 42 days [19]. For this study, some adaptations have been made to the original protocol: before the chronic treatment with sodium selenite or selenomethionine, the cells were pre-incubated (day −3 to 0) and the whole protocol was shortened to 35 days (Fig. 1). For the pre-incubation phase, untransformed BALB/c cells were seeded into 10-cm dishes (300,000 cells/dish) and treated with 0 µM, 0.01 µM, 0.1 µM or 1 µM sodium selenite or selenomethionine for 72 h (days −3 to 0) (Fig. 1). Subsequently, preincubated cells were harvested using trypsin/EDTA (Thermo Fisher Scientific), counted, and seeded into 6-well plates (2,500 cells/ml) as well as into 10-cm dishes (2,500 cells/ml) (day 0). For 6-well plates, 4 technical replicates were seeded. Cells were incubated with different concentrations of sodium selenite or selenomethionine (0, 0.01, 0.1, 1 µM) during the complete transformation assay (days 0 to 35). The cell culture medium was replaced by fresh medium supplemented with or without selenium every 3 to 4 days and with 0.5 µg/ml MCA (Sigma) (days 1 to 4) or 0.3 µg/ml TPA (Merck) (days 8 to 21), respectively. After 35 days, the BALB-CTA was terminated. Cells in 6-well plates were fixed with methanol and stained with basophilic giemsa solution (AppliChem). Type III foci of malignantly transformed cells were counted by two independent persons as described [20]. All cells in 10-cm dishes were completely harvested (*i.e.* all cells present at the dish without separating untransformed cells grown as a monolayer and transformed cells grown as foci) using trypsin/EDTA as described before.

### Cell Viability Assays

The effect of sodium selenite and selenomethionine on cell viability was measured with an MTT-assay. Cells were seeded into a 96-well plate as technical triplicates (5,000 cells/well) and treated with sodium selenite or selenomethionine for 72 h. Subsequently, the cell culture medium was discarded, and cells were incubated with FCS-free RPMI medium (without the addition of selenium) supplemented with 3-(4,5-Dimethyl-2-thiazolyl)−2,5-diphenyl-2*H*-tetrazoliumbromid (MTT; 0.5 mg MTT/ml; Merck), 1% P/S (Thermo Fisher Scientific) and 1.1% GlutaMAX™ (Thermo Fisher Scientific) for 120 min at 37 °C. Finally, the MTT-medium was replaced by dimethylsulfoxide (DMSO; Merck) and cell plates were shaken (600 rpm) for 10 min at RT to dissolve MTT formazan crystals. Absorption was measured at 550 nm and 690 nm (SynergyH1, Agilent Technologies) and the capacity of MTT reduction was calculated in relation to untreated control cells.

For quantification of viable cells, cells were seeded in a 6-well plate (25,000 cells/well) and treated with sodium selenite or selenomethionine. Twice a day (7, 24, 31, 47, 54, 70, 78, 96 h after seeding), adherent cells were harvested using trypsin/EDTA. The numbers of viable cells were counted using trypan blue (Merck) and an automated cell viability analyzer (Vi-Cell XR, Beckman Coulter, Brea, California, USA). To generate curves, the number of viable cells/ml was plotted against time.

### Cell Lysate Preparation

For enzyme activity assays, cell pellets were lysed in homogenization buffer (100 mM Tris (PanReac AppliChem, Darmstadt, Germany), 300 mM KCl (PanReac AppliChem), 0.1% triton X-100 (Carl Roth, Karlsruhe, Germany), 0.1% protease inhibitor cocktail set (Merck)) using a TissueLyser II (Qiagen, Venlo, Netherlands) for 2 × 30 s (30 Hz) followed by a centrifugation step for 10 min (15,000 × *g*, 4 °C). The supernatant was kept on ice and directly used for the measurement of NAD(P)H quinone dehydrogenase 1 (NQO1) enzyme activity before storing at −20 °C for further utilization.

For immunoblots and total reflection X-ray fluorescence (TXRF) measurements, whole cell lysates were prepared using radioimmunoprecipitation assay buffer (RIPA buffer; 50 mM Tris (PanReac AppliChem), 150 mM NaCl (Carl Roth), 2 mM EDTA (PanReac AppliChem), 0.5% Na desoxycholate (Merck), 0.1% SDS (PanReac AppliChem), 1% NP-40 Alternative (Merck), 0.1% protease inhibitor cocktail set (Merck)) by shaking for 15 min (1,200 rpm, 4 °C), sonification for 10 s (80% amplitude, 0.5 cycle), and centrifugation for 10 min (14,000 × *g*, 4 °C). Protein concentrations of cell lysates were quantified by a Bradford assay with Coomassie Brilliant Blue G-250 (BioRad, Hercules, California, USA) and bovine serum albumin (BSA; PanReac AppliChem) to generate a standard curve.

### Enzyme Activity Assays

Enzyme activity assays were conducted using homogenization buffer cell lysates, 96-well plates, and a microplate reader (SynergyH1, Agilent Technologies). Samples were measured as technical triplicates. Methods for GPX, TXNRD [21], and NQO1 [22] activity have been described before. For total GPX activity, a glutathione reductase-coupled NADPH-consuming assay was conducted with small changes within the procedure: briefly, assay reagents were preincubated with the sample for 15 min at 30 °C. The reaction was initiated by the addition of H_2_O_2_ (Merck) as substrate for GPXs (final concentration 50 µM). Absorbance was measured at 340 nm to monitor consumption of NADPH. TXNRD activity was estimated by a NAPDH-dependent reduction of 5,5’-dithiobis(2-nitrobenzoicacid) (DTNB; Merck) to 5’-thionitrobenzoate (TNB) that was quantified by an increase in absorbance at 412 nm. The NQO1 enzyme activity assay was based on a NQO1-catalyzed reduction of menadione (Merck) to menadiol that subsequently reacted with MTT (Merck). The increase of reduced MTT was measured at 590 nm. As the absorbance reader corrects filling levels to a light path length of 1 cm, enzyme activities can be calculated according to Lambert–Beer’s law. Enzyme activities were normalized to protein content analysed by Bradford assay.

### Immunoblot

To prepare Laemmli samples, 20–30 µg protein of homogenization buffer lysates or RIPA buffer lysates were used. 5 × Laemmli buffer (0.2085 M Tris (PanReac AppliChem) pH 6.8, 10% SDS (PanReac AppliChem), 50% glycerine (Carl Roth), 12.5% mercaptoethanol (Merck), 0.625% bromphenol blue (Carl Roth)) was mixed with the lysate to yield a 1/5 dilution of Laemmli buffer within the sample and heated for 5 min at 95 °C. Equal amounts of total protein were loaded onto SDS polyacrylamide gels, separated and transferred to nitrocellulose membranes. Subsequently, membranes were shaken in ponceau S solution (0.2% ponceau S (Carl Roth) in 3% trichloroacetic acid (Carl Roth)) for 2 min and protein bands were recorded by ChemiDoc™ MP Imaging System (Bio-Rad, Hercules, California, USA) and Image Lab 5.0 software. Next, membranes were blocked in 5% non-fat dry milk diluted in Tris-buffered saline containing 5 mM Tris (PanReac AppliChem), 15 mM NaCl (Carl Roth), 0.1% TWEEN® 20 (Merck) (T-TBS) for 1 h at RT. Primary antibody incubation took place over night at 4 °C with the following antibodies: rabbit GPX1 (1:10,000, Abcam, Cambridge, UK, ab108427), rabbit SELENOH (1:10,000, Abcam, ab151023), rabbit TXNRD1 (1:10,000, Abcam, ab124954), rabbit NQO1 (1:4,000, Abcam, ab34173), rabbit GPX4 (1:10,000, Abcam, ab125066), rabbit SELENOO (1:2,500, Abcam, ab172957), rabbit SELENOF (1:3,000, Abcam, ab124840), rabbit SELENOW (1:1,000, Genetex (Irvine, California, USA), GTX48717). Subsequent incubation with horseradish peroxidase-conjugated goat anti-rabbit IgG (1:50,000, Cell Signaling Technology, Danvers, Massachusetts, USA, 7074S) occurred for 1 h at RT. For chemiluminescent detection of protein bands, membranes were incubated in SuperSignal™ West Dura substrate (Thermo Fisher Scientific) for 1 min at RT, followed by imaging using ChemiDoc™ MP Imaging System (Bio-Rad). Band intensities were densitometrically quantified using the Image J software. Expression of target proteins was normalized to ponceau staining.

### Total Reflection X-ray Fluorescence Spectroscopy

To analyze intracellular trace element concentrations, the method of total reflection X-ray fluorescence (TXRF) spectroscopy was used. RIPA buffer lysates were mixed with an yttrium solution (final concentration 1 mg/l; Merck) that served as an internal standard for quantification. Quartz glass sample carriers (Bruker Nano GmbH, Berlin, Germany) were siliconized, loaded with 10 µl of sample-standard mixture, and dried at 40 °C. Each sample was placed twice as technical replicates and measured for 1,000 s with a TXRF spectrometer (Bruker S4 T-STAR, Bruker). Trace element concentrations were normalized to protein content for quantification.

### Statistics

Statistical calculations were performed using Graph Pad PRISM v9.5.1. Data are depicted as mean ± standard deviation. Single values of independent biological replicates are indicated as dots (*n* = 3–4). Differences between groups were analyzed using one-way ANOVA, two-way ANOVA or three-way ANOVA with following Bonferroni post-hoc test for multiple comparison. All statistical analyses were considered significant when *p* < 0.05.

## Results

### Establishment of Suboptimal, Adequate, and Supranutritional Treatment Conditions of Selenite And Selenomethionine in BALB/c Cells

**Fig. 2:**
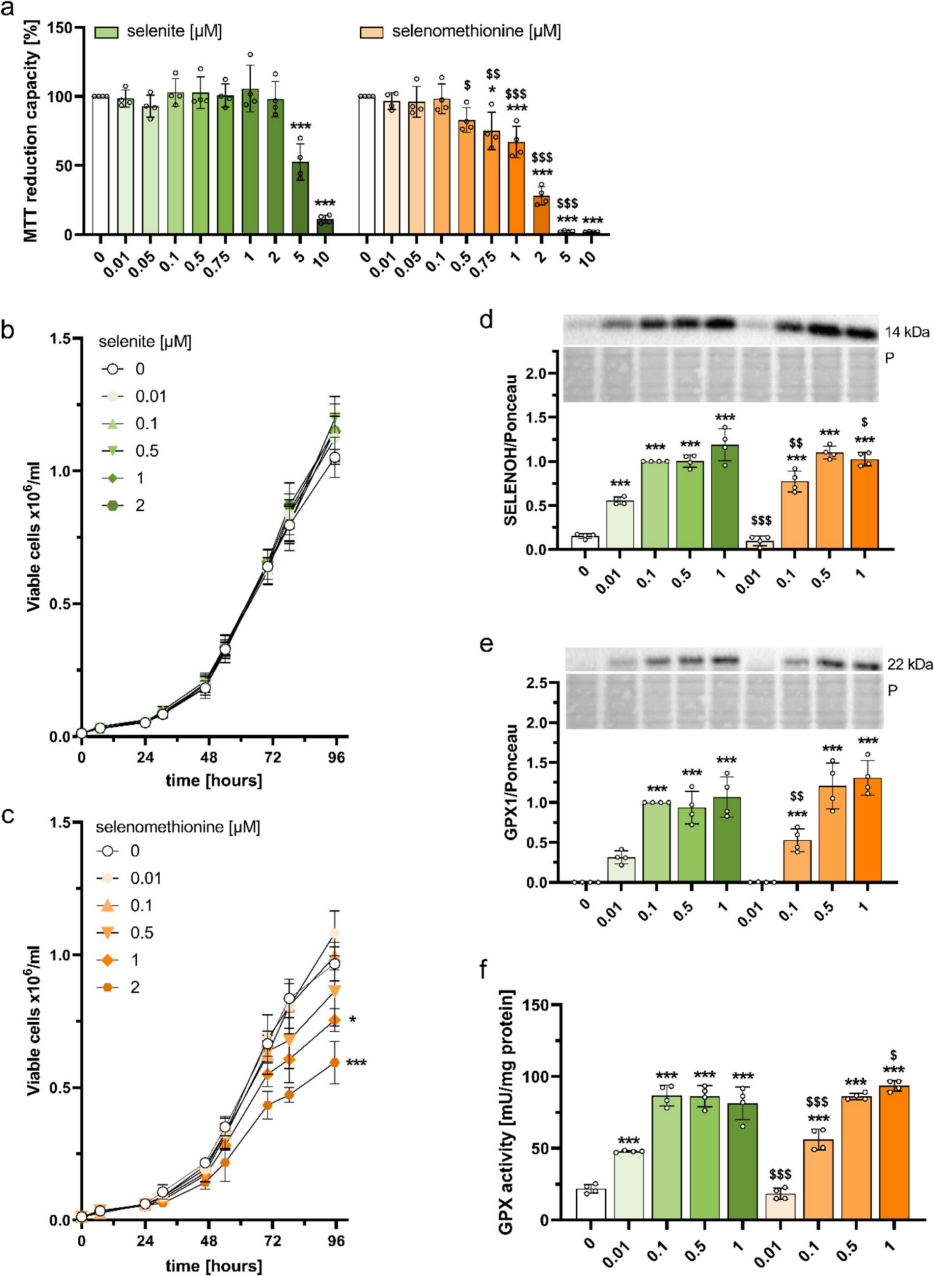
Identification of suboptimal, adequate, and supranutritional concentrations of selenite and selenomethionine in untransformed BALB/c cells. Starting with seeding, untransformed BALB/c cells were treated for 72 h (**a**, **d**-**f**) or up to 96 h (**b**-**c**) with increasing concentrations of sodium selenite or selenomethionine as indicated. MTT reduction capacity was compared to untreated cells by measuring absorbance of dissolved MTT formazan crystals (**a**). Viable cells were counted over 96 h after trypan blue staining (**b**, **c**). Protein expression of SELENOH (**d**) and GPX1 (**e**) were determined by Western blotting and normalized to ponceau staining. GPX activity was measured using a glutathione reductase-coupled NADPH-consuming assay and normalized to protein content of the cell lysates (**f**). Results are presented as mean ± SD (*n* = 4). Biological replicates are indicated by individual dots (**a**, **d**-**f**). Statistical analyses were based on two-way ANOVA with Bonferroni’s post-test (**a**, **d**-**f**) or one-way ANOVA with Bonferroni’s post-test for the 96-h time point (**b**, **c**). *p < 0.05, ***p < 0.001 vs. 0 µM; ^$^*p* < 0.05; ^$$^*p* < 0.01; ^$$$^*p* < 0.001 vs. selenite

To identify non-cytotoxic concentrations of selenium species for a chronic treatment of BALB/c cells within the BALB-CTA, untransformed BALB/c cells were treated with increasing concentrations of sodium selenite or selenomethionine up to 10 µM for 72 h. Treatment with up to 2 µM selenite had no effect on cellular viability measured by MTT reduction capacity (Fig. 2a). In contrast, cells treated with selenomethionine showed a concentration-dependent decline in MTT reduction capacity that was already detectable at 0.75 µM, with 62% MTT reduction capacity in comparison to the untreated control (Fig. 2a). Accordingly, there were significant differences between both selenium species starting at a concentration of 0.5 µM. In addition to the MTT assay, the number of viable cells was analysed for up to 96 h by counting cells after trypan blue staining. Again, there were no effects of selenite for concentrations up to 2 µM (Fig. 2b), whereas there was a significant decline in the number of viable cells after treatment with 1 and 2 µM selenomethionine (Fig. 2c). This indicates that higher concentrations of selenomethionine reduced both the metabolic activity (Fig. 2a) and the number of viable cells (Fig. 2c) probably primarily via inhibiting cellular proliferation and not via direct cytotoxic effects. Thus, concentrations of up to 1 µM were chosen for further experiments for both selenium species.

Next, we aimed to define treatment conditions that resulted in a differential upregulation of selenoproteins modulated by the selenium supply. Therefore, untransformed BALB/c cells were treated with selenite or selenomethionine with increasing concentrations up to 1 µM for 72 h and protein levels and enzyme activities of selenoproteins were detected. Both selenium species upregulated the protein expression of SELENOH and glutathione peroxidase 1 (GPX1) in a concentration-dependent manner but there was no increase of selenoprotein expression by 0.01 µM selenomethionine (Fig. 2d, e). Notably, cells treated with selenite reached a saturation of selenoprotein expression at lower concentrations than selenomethionine treated cells (0.1 µM selenite vs. 0.5 µM selenomethionine). The total GPX activity showed a selenium responsiveness that was comparable to that of GPX1 protein levels (Fig. 2f). Consequently, we defined 0.01 µM, 0.1 µM and 1 µM as suboptimal, adequate, and supranutritional concentrations for the treatment of BALB/c cells with both selenium species.

### Chronic Treatment with a Supranutritional Concentration of Selenomethionine Decreased Malignant Cell Transformation while Selenite had no Effect

**Fig. 3:**
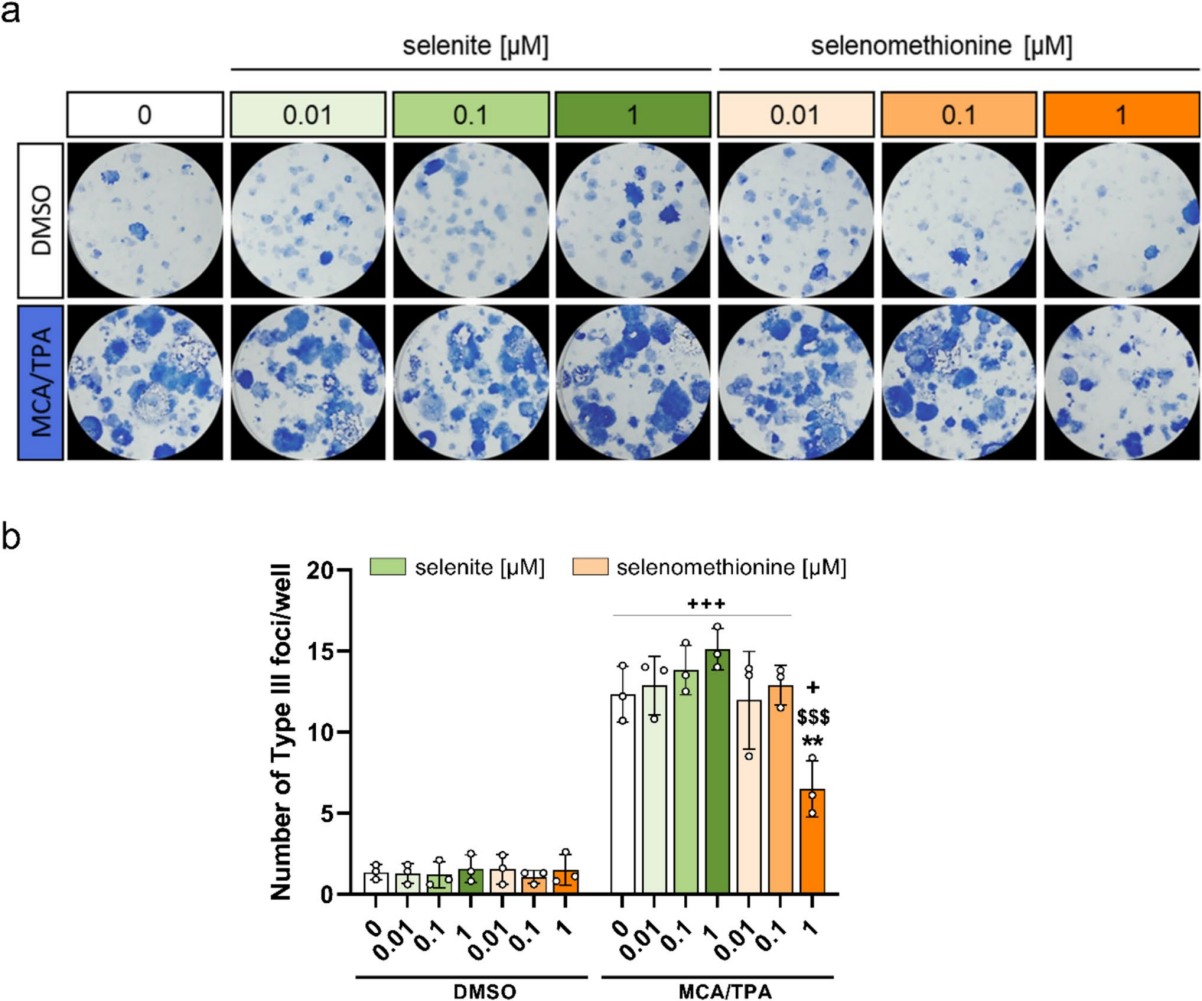
Chronic treatment with suboptimal, adequate, or supranutritional concentrations of selenite and selenomethionine within the BALB-CTA. Representative pictures of fixed and trypan blue stained cells are shown (**a**). Type III foci of malignantly transformed cells were counted according to specific phenotypic criteria by two persons independently and the mean value was calculated (**b**). Results are presented as mean ± SD. Biological replicates are indicated by individual dots (n = 3). Statistical analyses were based on three-way ANOVA with Bonferroni’s post-test. **p < 0.01 vs. 0 µM; ^$$$^*p* < 0.001 vs. selenite; ^+^*p* < 0.05, ^+++^*p* < 0.001 vs. DMSO. MCA: 3-methylcholanthrene; TPA: 12-O-tetradecanoyl-phorbol-13-acetate

To investigate the effects of selenium in a concentration- and species-specific manner on the neoplastic transformation, a BALB-CTA was conducted. BALB/c cells were treated with selenite or selenomethionine at suboptimal, adequate, or supranutritional concentrations (*i.e.,* 0.01 µM, 0.1 µM, and 1 µM) (Fig. 1). The cultivation of untransformed BALB/c cells with selenium species started 72 h prior to their seeding into the BALB-CTA and continued over the whole assay duration as a chronic treatment with medium refreshment every 3–4 days. Thus, differences in selenoprotein expression among the treatment conditions were already achieved before treating the cells with MCA as tumor initiator. After 5 weeks, the cell layers were fixed with methanol and stained with trypan blue visualizing cell foci formation (Fig. 3a). As expected, there were almost no Type III foci under conditions without MCA/TPA treatment which was independent of the selenium treatment. However, treating cells with MCA/TPA resulted in Type III foci development. Only the supranutritional concentration of selenomethionine significantly decreased the number of malignantly transformed foci compared to MCA/TPA-treated cells without selenium (6.5 ± 1.7 vs. 12.3 ± 1.7 type III foci per well, p < 0.01) (Fig. 3b). Neither suboptimal nor adequate concentrations of selenomethionine affected malignant cell transformation within the BALB-CTA (Fig. 3b). In contrast to selenomethionine, chronic treatment with supranutritional selenite appeared to increase the number of malignantly transformed foci in comparison to cells without additional selenium (15.1 ± 1.3 vs. 12.3 ± 1.7 type III foci per well), though this effect was not significant (Fig. 3a + b). Also, suboptimal and adequate selenite concentrations did not affect the number of type III foci in the BALB-CTA.

During the BALB-CTA, representative pictures of the cell layers of the different treatment conditions were taken. There were no differences between all conditions on day 1 after seeding. Only the combined treatment with MCA/TPA and supranutritional selenomethionine resulted in fewer cells which was visible at day 6 after MCA treatment and before TPA treatment as well as on day 12 during TPA treatment (Fig. S1). Until the end of the assay (day 35, assessment of type III foci), the cell monolayer of untransformed cells surrounding the foci was fully confluent in all wells independent of the treatment condition.

### Selenium Concentrations and Selenoprotein Expression were Increased by both Selenium Species Independent of MCA/TPA Treatment

**Fig. 4:**
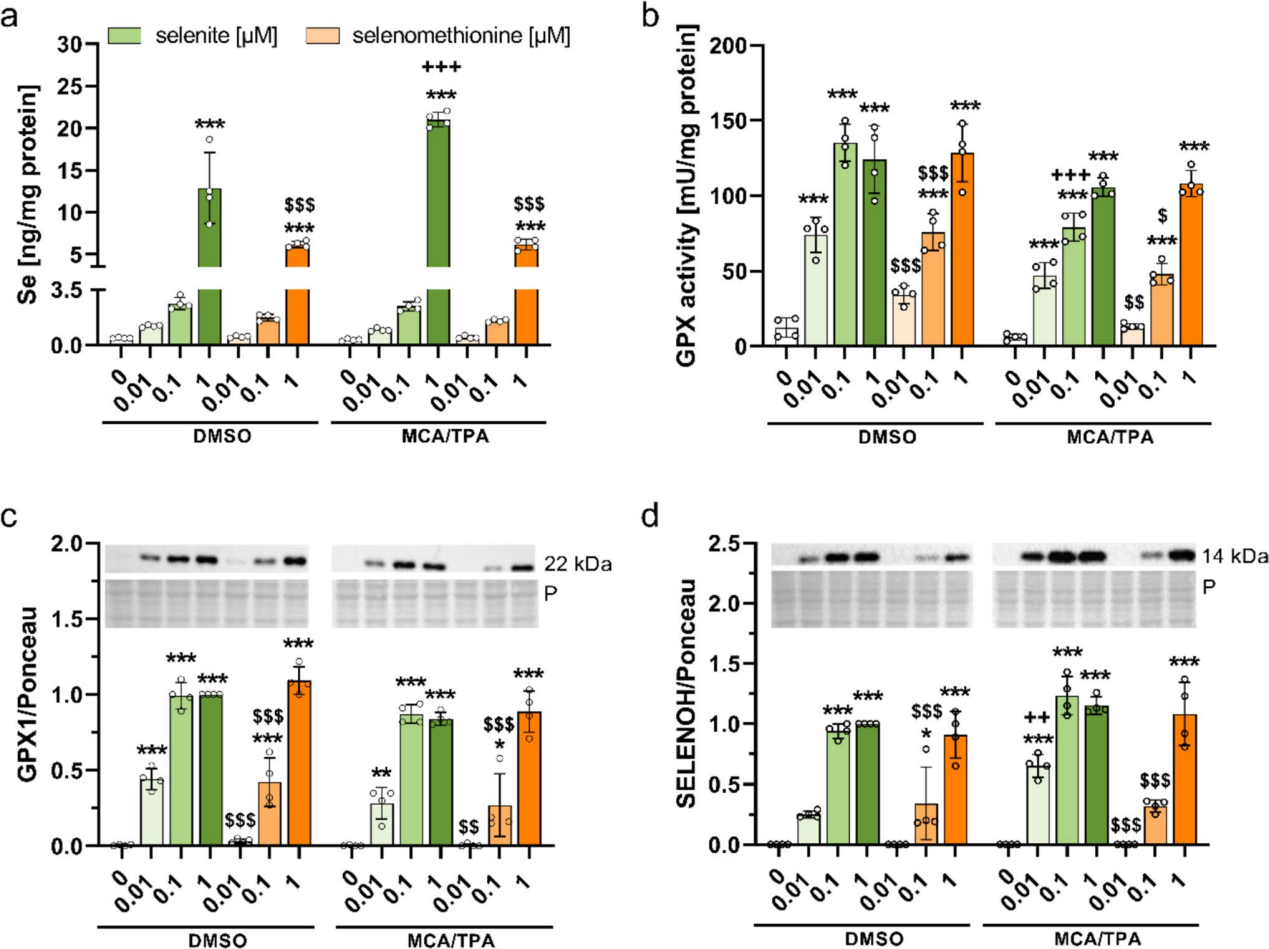
Selenium status of BALB/c cells after chronic treatment with selenite or selenomethionine within the BALB-CTA. Cells were harvested after completed BALB-CTA and lysates were prepared for further analysis. Intracellular concentrations of selenium were analyzed by total reflection X-ray fluorescence (TXRF) with 1 mg/L Yttrium as standard for 1000 s and normalized to protein content of the cell lysates (**a**). GPX activity was measured using a glutathione reductase-coupled NADPH-consuming assay and normalized to protein content of the cell lysates (**b**). Protein expression of GPX1 (**c**) and SELENOH (**d**) were determined by Western blotting and normalized to ponceau staining. Results are presented as mean ± SD. Biological replicates are indicated by individual dots (n = 4). Statistical analyses were based on three-way ANOVA with Bonferroni’s post-test. ***p < 0.001 vs. 0 µM; ^$^*p* < 0.05; ^$$^*p* < 0.01; ^$$$^*p* < 0.001 vs. selenite; ^++^*p* < 0.01, ^+++^*p* < 0.001 vs. DMSO

Next, we aimed to characterize the selenium status of MCA/TPA- or DMSO-treated cells in response to the two selenium species using whole cell lysates representing a mixture of all cellular stages present at the cell dish at the end of the BALB-CTA. There was a clear concentration difference for each selenium species with highest intracellular selenium concentrations when supplying supranutritional concentrations (Fig. 4a). Selenite treatment resulted in higher selenium concentrations than selenomethionine, which was most pronounced under supranutritional conditions (DMSO: 12.9 ± 4.2 vs. 6.2 ± 0.4 ng/mg, *p* < 0.001; MCA/TPA: 21.0 ± 0.9 vs. 6.2 ± 0.6 ng/mg, *p* < 0.001). Interestingly, MCA/TPA-treated cells accumulated more selenium compared to DMSO-treated cells when supplied with a supranutritional selenite concentration (21.0 ± 0.9 vs. 12.9 ± 4.2 ng/mg, *p* < 0.001).

In line with the intracellular selenium concentrations, both selenium species increased the GPX activity and protein expression of GPX1 and SELENOH in a concentration-dependent manner, reaching similar maximum values (Fig. 4b-d). The selenite-treated cells had higher selenium concentrations than the selenomethionine-treated cells and also higher levels of all three selenoprotein markers at concentrations of 0.01 and 0.1 μM. Accordingly, selenite-treated cells reached maximum values already at lower concentrations than selenomethionine-treated cells (0.01 µM vs. 1 µM). This effect was equally observed in DMSO- and MCA/TPA-treated cells. However, some differences between DMSO- and MCA/TPA-treated cells were also observed for the selenoprotein expression with overall lower GPX1 expression and GPX activity in MCA/TPA-treated cells compared to DMSO-treated cells (overall effect *p* < 0.001) but higher SELENOH expression (MCA/TPA-groups vs. DMSO-groups, overall effect *p* < 0.001). We observed a similar concentration-dependent increase in selenoprotein expression after treatment with both selenium species for SELENOW and SELENOF (Fig. S2a, b). Interestingly, the increase in selenium concentration of MCA/TPA-treated cells with a supranutritional selenite concentration was not reflected on the level of selenium-sensitive selenoprotein expression.

**Fig. 5:**
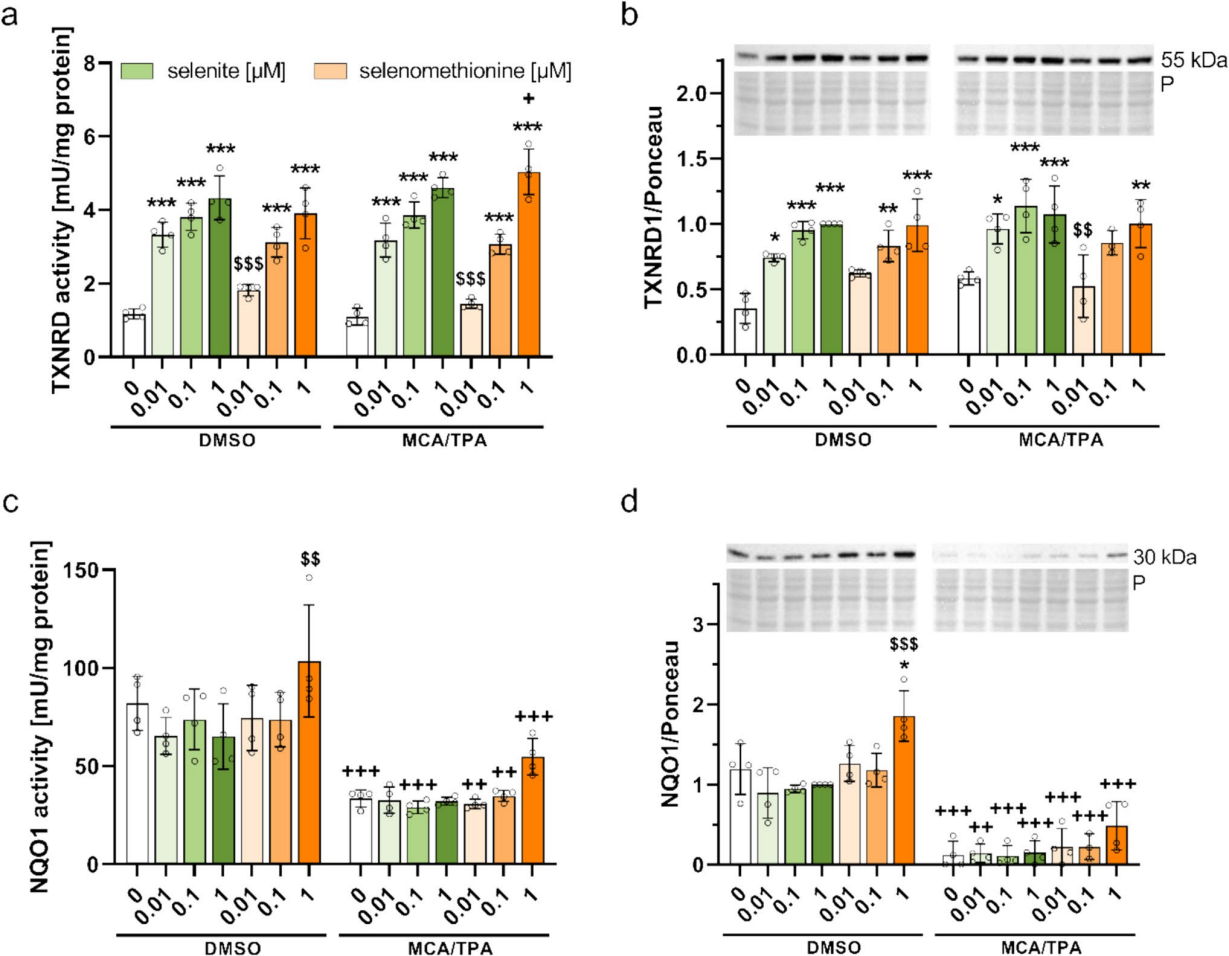
Activity and protein expression of NRF2-regulated proteins in BALB/c cells after chronic treatment with selenite or selenomethionine within the BALB-CTA. Cells were harvested after completed BALB-CTA and lysates were prepared for further analysis. TXNRD activity was analyzed by an NADPH-dependent reduction of DTNB to TNB and normalized to protein content of the cell lysates (**a**). Protein expression of the selenoprotein TXNRD1 (**b**) and of NQO1 (**d**) were determined by Western blotting and normalized to ponceau staining. NQO1 activity was measured by a menadione-mediated reduction of MTT and normalized to protein content of the cell lysates (**c**). Results are presented as mean ± SD. Biological replicates are indicated by individual dots (*n* = 4). Statistical analyses were based on three-way ANOVA with Bonferroni’s post-test. *p < 0.05, **p < 0.01, ***p < 0.001 vs. 0 µM; ^$$^*p* < 0.01; ^$$$^*p* < 0.001 vs. selenite; ^+^*p* < 0.05, ^++^*p* < 0.01, ^+++^*p* < 0.001 vs. DMSO

To further characterize the selenium status of the cells, TXNRD activity and TXNRD1 expression as well as GPX4 expression were included as important representatives of selenoproteins which are less sensitive towards changes in the selenium supply. In line with this, TXNRD activity and protein expression of TXNRD1 (Fig. 5a, b) and GPX4 as well as SELENOO (Fig. S2c, d) showed a similar pattern of selenium responsiveness as selenium-sensitive selenoproteins, but differences were less pronounced. Only the treatment with 0.01 µM selenite or selenomethionine revealed a significant difference between the selenium species. Remarkably, the supranutritional concentration of selenomethionine increased TXNRD activity only in transformed cells compared to untransformed cells (5.0 ± 0.6 vs. 3.9 ± 0.7 mU/mg protein, *p* < 0.05) (Fig. 5a), while there were no differences between transformed and untransformed cells for the other selenoproteins under the same treatment condition.

### The Supranutritional Concentration of Selenomethionine Upregulates NRF2 Target Genes

As TXNRD1 is regulated by the redox-sensitive transcription factor nuclear factor erythroid 2-related factor 2 (NRF2), we additionally analyzed NQO1 which is one of the prototypical NRF2 target genes widely used to indicate the activity of the NRF2 pathway. NQO1 enzyme activity and protein expression were strongly decreased in MCA/TPA-treated cells compared to DMSO-treated cells which was independent of the selenium supply (Fig. 5c, d). Notably, treatment with the supranutritional concentration of selenomethionine increased NQO1 activity and protein expression in untransformed and transformed cells compared to cells without selenium, an effect that was not observed for selenite, suggesting that NQO1 might be upregulated by a high concentration of selenomethionine.

## Discussion

In the present study, we aimed to use a well-established murine in vitro model (BALB-CTA) to specifically address the role of the selenium supply as well as of different selenium species during early phases of malignant cell transformation starting from untransformed cells. We used selenomethionine and selenite as selenium sources, as these are of physiological importance to improve the selenium status in humans. Worldwide, there is a broad variety of nutritional selenium uptake ranging from deficient to high concentrations [23]. For example, Europeans have a suboptimal selenium status characterized by not yet optimized selenoprotein expression [4]. Therefore, the objective of this study was to investigate several physiological relevant concentrations and species for the selenium supply.

Our results demonstrated that both selenium species increased the activity and expression of all investigated selenoproteins (including GPX1, SELENOH, SELENOW, SELENOF, TXNRD1, GPX4, and SELENOO) in a concentration-dependent manner leading to similar maximal values. This effect was comparable between DMSO- and MCA/TPA-treated cells, *i.e.,* between untransformed and transformed cells. However, the two selenium species differed in their ability to increase intracellular selenium concentrations and accordingly selenoprotein expression (Fig. 4). In cell culture, selenite is the most commonly used selenium species. In line with our results, previous cell culture studies have also detected a stronger response of selenoprotein activity and protein expression to selenite than to selenomethionine [21, 24]. This can be attributed to various transporters for selenite and selenomethionine, including XCT for selenite uptake, especially in tumor cells [25]. In addition, both selenium species have a different capacity to serve as a source for selenoprotein biosynthesis due to different metabolic conversion rates and pathways which in the case of selenomethionine is related to methionine metabolism and concentration [21].

An optimization of selenoprotein expression as a result of an adequate supply with selenium is discussed to be protective in early phases of carcinogenesis due to an improved antioxidant defence and increased protection against DNA damage [10]. Surprisingly, neither a suboptimal nor an adequate chronic supply with selenium in form of selenite or selenomethionine had any effect on type III foci development in the BALB-CTA (Fig. 3a, b). This indicates that enhanced expression of several selenoproteins in parallel does not affect the end point (type III foci development) in the BALB-CTA after 38 days of intervention. This contrasts with previous papers, describing tumor promoting or suppressing effects of individual selenoproteins. While SELENOH upregulation suppressed tumor cell proliferation [26], SELENOF is supporting tumor formation [27]. Hence, the outcome of an upregulation of individual selenoproteins in terms of pro- or anti-tumorigenic effects might be overwritten by an increased expression of the whole selenoproteome. Independent from the selenium supply, we observed general differences in enzyme activity and protein expression patterns between DMSO- and MCA/TPA-treated cells. Our data indicated that GPX enzyme activity and GPX1 protein expression levels were downregulated, while SELENOH protein expression was increased in MCA/TPA-treated cells compared to DMSO-treated cells (Fig. 4b-d). However, both selenoproteins have been shown to be upregulated in several types of tumors, and especially high GPX1 levels are associated with poor prognosis [26, 28]. An upregulation of GPX2 within transformed cells, as described for different tumor tissues [8], could not be confirmed as this epithelial-localized isoform was not detectable within the mesenchymal BALB/c cells. Overall, there was no net selenoprotein effect on malignant cell transformation when considering several selenoproteins in parallel and in response to a modulated selenium supply.

Only the treatment with the supranutritional concentration of selenomethionine resulted in a reduced number of type III foci compared to MCA/TPA-treated cells without selenium, demonstrating a reduced malignant cell transformation and a protective effect (Fig. 3a, b). Thus, selenomethionine present at non-cytotoxic concentrations appears to have preventive effects that are independent of selenoproteins. The supranutritional concentration of selenomethionine inhibited BALB/c cell proliferation (Fig. 2c) which was also observed during the BALB-CTA (Fig. S1). In several human cancer cell lines selenomethionine exerts a time- and dose-dependent inhibition of cell growth [29, 30], as well as a transcriptional downregulation of genes involved in cell cycle [31]. Consequently, our observed protective effects could either be directly mediated by selenomethionine itself or indirectly by metabolites derived from selenomethionine. High concentrations of selenomethionine can be directly metabolized to methylselenol by the murine selenomethionine-α,γ-elimination enzyme [32, 33]. A cotreatment of mice with selenomethionine and the selenomethionine-α,γ-elimination enzyme led to a decreased tumor growth and prolonged survival in a tumor xenograft model [34]. Methylselenol is supposed to have preventive effects and to induce apoptosis [35, 36] which could result from interactions between methylselenol and disulfide bonds of redox-sensitive proteins [36]. This way, selenomethionine can increase NQO1 protein expression in human cell lines [37]. Our results also indicate an upregulation of NQO1 activity and protein expression in BALB/c cells treated with high concentrations of selenomethionine (Fig. 5c, d) which might provide an explanation for the observed tumor suppressive effect. NQO1 is a phase II enzyme with multiple cellular functions including the detoxification of electrophilic quinones and thereby preventing redox cycling reactions and the generation of ROS, or the stabilization of target proteins like the tumor suppressor protein p53 via binding and protecting against proteasomal degradation [38]. NQO1 expression is regulated by the transcription factor NRF2 [39] which has been shown to be activated under high selenium conditions [40]. NRF2 is discussed to have a preventive role in cancer, as its activation is followed by the transcription of a multitude of target genes including NQO1 and TXNRD1 that are involved mainly in antioxidant protection and detoxification of ROS [41]. However, a cancer-preventive effect of NRF2 activation is restricted to early stages of carcinogenesis while it promotes malignant progression examined in chemical-induced carcinogenesis and xenograft tumor models [42, 43]. Therefore, the observed preventive effect of a chronic supply with the supranutritional concentration of selenomethionine within the BALB-CTA might result from the transcription of NRF2 target genes already in early stages of *in vitro* carcinogenesis.

In contrast to high levels of selenomethionine, the supranutritional concentration of selenite showed a trend for an increased number of malignantly transformed type III foci in the BALB-CTA and hence might increase malignant cell transformation (Fig. 3a, b). Interestingly, treatment with the supranutritional selenite concentration increased intracellular selenium concentrations of BALB/c cells to a higher extend than the same treatment concentration of selenomethionine and the effect was stronger in transformed than in untransformed cells (Fig. 4a). Cancer cells are selenophilic, *i.e.,* they have an increased uptake of selenite due to an increased expression of XCT transporters [44]. Supranutritional concentrations of selenite have pro-oxidant effects [45] and therefore, could induced oxidative stress supporting malignant cell transformation within the BALB-CTA. In contrast, other studies observed anti-cancer properties like the induction of apoptosis in cancer cells as well as a therapeutic effect in xenograft tumor models [46–48]. Accordingly, the timing of the intervention might be key: the supplementation of supranutritional doses of selenite during the promotion or progression of chemically induced-carcinogenesis in rat models decreased tumor incidence and number [49, 50] and tumor volume of pre-neoplastic liver nodules [51], while a supplementation during the initiation phase had no preventive effect or even increased liver nodule density [49–51]. Unfortunately, using the phase-specific approach in the BALB-CTA was limited by the fact that selenium supplied during initiation was maintained on a high level for further weeks even though it was eliminated from the medium.

## Conclusion

Within the BALB-CTA, a chronic supply with high selenomethionine concentrations exhibited preventive effects in contrast to high levels of selenite. This effect appears to be mediated by mechanisms other than upregulation of selenoproteins, since adequate selenium supply in the form of selenomethionine or selenite had no preventive effect. Overall, our results support the finding, that the chemical species as well as the dosage are crucial for the cancer prevention mediated by selenium.

## Supplementary Information

Below is the link to the electronic supplementary material.

Supplementary file1 (PDF 535 KB)

## Data Availability

No datasets were generated or analysed during the current study.
