# Supplementary material for: Supranutritional Selenomethionine but not Selenite Reduces Malignant Cell Transformation
Source: Biol Trace Elem Res. 2025 Jun 25;204(2):956–68. doi: 10.1007/s12011-025-04719-6 (PMC12847112; doi:10.1007/s12011-025-04719-6)
Supplement: Supplementary file 1 — Supplementary file1 (PDF 535 KB) [file 12011_2025_4719_MOESM1_ESM.pdf]

Supplementary information

Supranutritional selenomethionine but not selenite reduces malignant cell transformation

Caroline E. Meyer[1], Maria Schwarz[1], Felix B. Meyer[2], René Thierbach[2], Anna P. Kipp[1]

Affiliations:

[1]Nutritional Physiology, Institute of Nutritional Sciences, Friedrich Schiller University Jena

[2]Human Nutrition, Institute of Nutritional Sciences, Friedrich Schiller University Jena

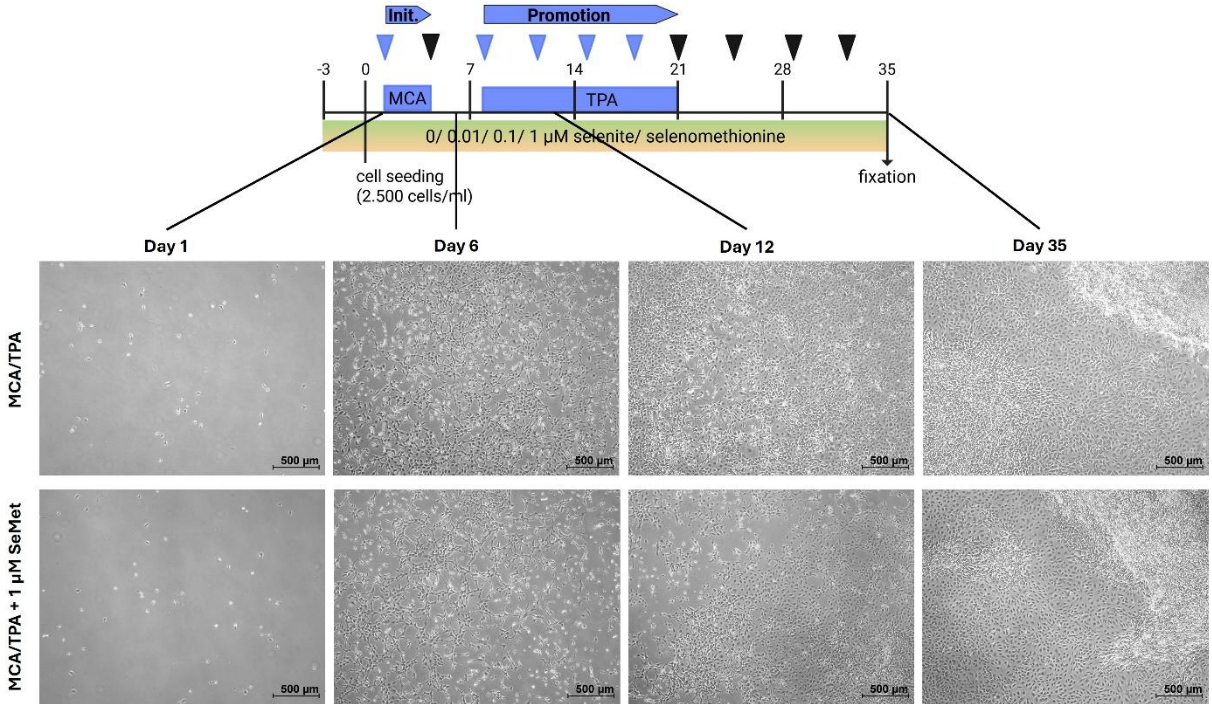

Figure S1: Representative pictures of the cell layers during the BALB-CTA. Microscopic images of the cell layer were taken at day 1, 6, 12, and 35 of the BALB-CTA. The scale bar corresponds to 500 µm. Created in BioRender. Lossow, K. (2025) https://BioRender.com/9tu7eu3

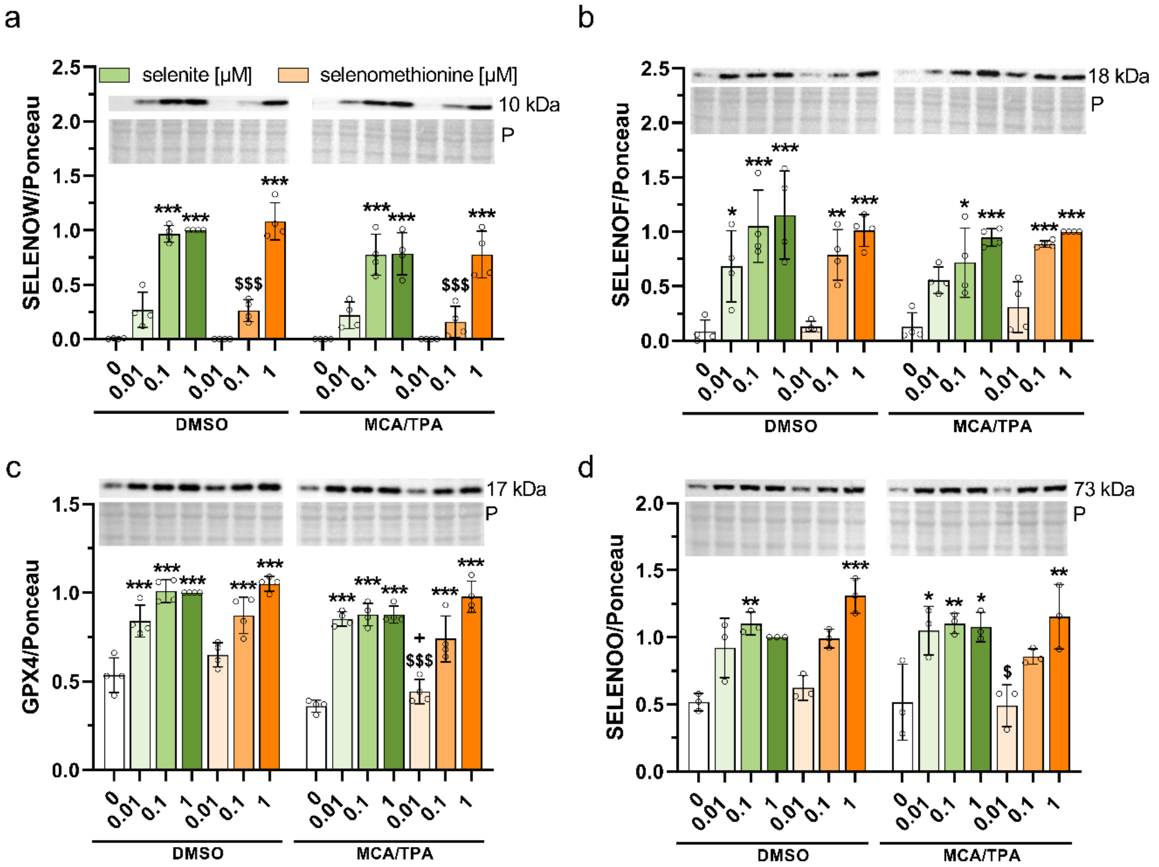

Figure S2: Protein expression of selenoproteins of BALB/c cells after chronic treatment with selenite or selenomethionine within the BALB-CTA. Cells were harvested after completed BALB-CTA and lysates were prepared for further analysis (a-d). Protein expression of the selenoproteins SELENOW (a), SELENOF (b), GPX4 (c), and SELENOO (d) was determined by Western Blot and normalized to ponceau staining. Results are presented as mean ± SD. Biological replicates are indicated by individual dots (n=4). Statistical analyses were based on three-way ANOVA with Bonferroni's post-test. ***p < 0.001 vs. 0 µM; $^{\$}p < 0.05$; $^{\$\$}p < 0.01$; $^{\$\$\$}p < 0.001$ vs. selenite; $^{++}p<0.01$, $^{+++}p<0.001$ vs. DMSO.
